# Higher-Than-Conventional Subcutaneous Regular Insulin Doses Following Diabetic Ketoacidosis in Children and Adolescents

**DOI:** 10.4274/jcrpe.3925

**Published:** 2017-06-01

**Authors:** Özlem Bağ, Selma Tunç, Özlem Nalbantoğlu, Çiğdem Ecevit, Aysel Öztürk, Behzat Özkan, Korcan Demir

**Affiliations:** 1 Dr. Behçet Uz Children’s Hospital, Clinic of Pediatrics, İzmir, Turkey; 2 Dr. Behçet Uz Children’s Hospital, Clinic of Pediatric Endocrinology, İzmir, Turkey; 3 Dokuz Eylül University Faculty of Medicine, Department of Pediatrics, Division of Pediatric Endocrinology, İzmir, Turkey

**Keywords:** type 1 diabetes mellitus, regular insulin, initial doses, children, adolescent

## Abstract

**Objective:**

To evaluate the effect of initial insulin dosage on glycemic control in the first 48 hours of subcutaneous regular insulin therapy after resolution of diabetic ketoacidosis (DKA).

**Methods:**

Records of patients with DKA hospitalized in the past 3 years [n=76, median age=10.0 (6.0-12.0) years, Male/Female: 44/32] were reviewed. The patients were designated into two groups according to distribution of starting doses of subcutaneous insulin. Group 1 (n=28) received a median dose of 1.45 U/kg/day (1.41-1.5) and group 2 (n=48) a median dose of 0.96 U/kg/day (0.89-1). Clinical and laboratory data were analyzed.

**Results:**

Median, minimum, and maximum blood glucose levels of Group 1 in the first 48 hours of treatment were significantly lower than that of Group 2 [213 (171-242) vs. 255 (222-316), p=<0.001; 102 (85-151) vs. 129 (105-199), p=0.004; and 335 (290-365) vs. 375 (341-438), p=0.001, respectively]. The number of patients who experienced hypoglycemia (<70 mg/dL) were similar [Group 1, 5 (17.9%) vs. Group 2, 4 (8.3%), p=0.276] and none had severe hypoglycemia. In Group 1, the ratio of blood glucose levels within the target range (100-200 mg/dL) were higher (37.5% vs. 12.5%) and the number of results >200 mg/dL were lower (50% vs. 81.3%) compared to Group 2 (p=0.001 and p<0.001, respectively).

**Conclusion:**

After resolution of DKA, a higher initial dose of 1.4-1.5 U/kg/day regular insulin is associated with better glycemic control in children and adolescents without an increase in risk of hypoglycemia.

## What is already known on this topic?

Although dosages of 0.5 U/kg/day to 2 U/kg/day have been used as initial doses in various diabetes centers, little is known about their effect on glycemic control in new-onset type 1 diabetes mellitus (T1DM).

## What this study adds?

An initial dose of 1.4-1.5 U/kg/day regular insulin may safely be used after resolution of diabetic ketoacidosis in children with new-onset T1DM without an increased risk of hypoglycemia.

## INTRODUCTION

Diabetic ketoacidosis (DKA) occurs in 20-40% of children and adolescents with new-onset type 1 diabetes mellitus (T1DM) and after DKA resolves, the therapy is switched to any insulin regimen that aims to control blood glucose (BG) levels. It is known that the required initial daily insulin dose may vary according to many factors including age, body weight, stage of puberty, duration and phase of diabetes (1). The optimal insulin dose for the patient can only be determined empirically (2). An excellent initial insulin dose estimate is one that provides tight BG control and minimizes the risk of hypoglycemia.

Regular insulin is a soluble crystalline zinc insulin, an essential component of most daily replacement regimens (3). Due to its chemical structure, it has a wide peak and a long tail for bolus insulin, and thus cannot mimic the activity of the β cell but is available to initiate treatment after resolution of DKA and serves to determine the daily insulin dose before basal-bolus insulin regimen. Although guidelines recommend 0.5-1.0 U/kg/day of subcutaneous insulin following resolution of DKA, up to 2 units/kg/d are used in various centers, depending on the preference and experience of the particular diabetes team.

It has previously been reported that intensive insulin therapy would improve endogenous insulin secretion, consequently leading to better metabolic control (4). Thus, one of the aims of therapy following DKA is to control BG levels as early as possible. Higher initial insulin doses could rapidly decrease BG level, but their effect on BG fluctuations have not been extensively investigated (5). This present study aimed to evaluate the effect of the initial insulin dose on glycemic control in the first 48 hours of DKA treatment in children and adolescents with new-onset T1DM and also to compare BG fluctuations with higher and conventional doses of subcutaneous regular insulin therapy.

## METHODS

**Figure 1:**
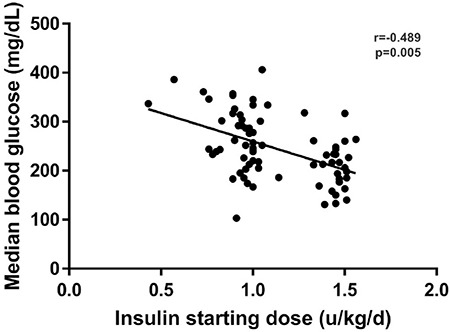
Distribution of the starting subcutaneous insulin doses and its correlation with median blood glucose levels during the first 48 hours of treatment

The study was conducted in one of the major tertiary hospitals in the region. Hospital records of patients who presented in the last 3 years were reviewed for the study. Diagnosis of T1DM and DKA were made according to the 2014 International Society for Pediatric and Adolescent Diabetes (ISPAD) Clinical Practice Consensus Guidelines for Diabetes in Childhood and Adolescence (6). Newborns, patients who had been treated with any insulin or antihyperglycemic drugs before admission, patients having additional endocrine (hypo-hyperthyroidism, hypo-hypercortisolism, etc.) or non-endocrine diseases (any infectious or inflammatory disease), and those with inadequate hospital records were excluded. Finally, 76 children and adolescents [median age=10.0 (6.0-12.0) years, Male/Female: 44/32] who presented with DKA due to new-onset T1DM were enrolled as the study group. Age, gender, stage of puberty (patients were noted as pubertal if they had at least Tanner 2 breast development or ≥4 mL testicular volume), body weight and height, glycosylated hemoglobin (HbA1c) levels, BG levels on admission and at the start of regular subcutaneous insulin, insulin dose administered for DKA, and initial dose of regular subcutaneous insulin were recorded. As shown in Figure 1, the patients were designated into two groups according to distribution of starting doses of subcutaneous insulin. Group 1 consisted of patients who received ≥1.25 U/kg/day [n=28, median dose=1.45 U/kg/day (1.41-1.5)] and Group 2 consisted of those who were treated with <1.25 U/kg/day [n=48, median dose=0.96 U/kg/day (0.89-1)].

Clinical and laboratory data were collected and analyzed after Behçet Uz Children’s Hospital Ethics Committee’s approval, in concordance with the principles of Declaration of Helsinki (7).

### Treatment Protocol

After resolution of DKA, all patients with new-onset T1DM were started on regular insulin (Humulin R, Lilly, USA) every 6 hours to determine the daily insulin requirement before transition to basal-bolus regimen. As the tissue half-life of insulin is longer than that of intravenous insulin, the first dose of subcutaneous basal insulin was given 30 min before the cessation of intravenous insulin infusion. Premeal BG measurement was performed before each insulin injection 30 minutes before the meals and the insulin dose was determined according to the BG levels: 100-200 mg/dL, same as the previous dose; >200 mg/dL, 110% of the previous dose; <100 mg/dL, 90% of the previous dose (2). The insulin dose was also adjusted according to the consumed amount of the meals. The decision to switch to basal-bolus regimen was made when no significant change was needed in regular insulin doses, generally after 3-4 days. BG levels were measured more frequently in patients who suffered from any symptom of hypo- or hyperglycemia. During hospitalization, the meals of the patients were prepared by dietitians according to the ISPAD Clinical Practice Consensus Guidelines 2014 on nutritional management in children and adolescents with diabetes. The diets contained carbohydrates providing approximately 50-55%, fat up to 30-35%, and protein 10-15% of daily energy requirements (8). Four meals and three snacks were given a day and no additional food was consumed unless hypoglycemic events occurred.

### Study Variables

Descriptive characteristics of the patients, treatment information, and every BG measurement during the first 48 hours of subcutaneous regular insulin treatment were recorded. Glycemic variability indices [standard deviation (SD), coefficient of variation (CV), maximum BG, minimum BG, difference between maximum and minimum BG, rate of BG change (the amount of change between consecutive measurements, mg/dL/min)] were calculated. All BG measurements were performed by the same capillary BG monitoring system (Astracheck Plus^®^, Medisign MM 600, Empecs, Beijing, China), an electrochemical glucometer using the modified glucose oxidase method, calibrated monthly by electronical calibrators.

### Statistical Analysis

The data were statistically analyzed using computer software SPSS 15.0 (Chicago, IL, USA). Mann-Whitney U-test and chi-square test were used to compare numerical and categorical variables, respectively, between groups. Univariate correlation analysis was performed between insulin starting dose and median glucose levels during 48 hours. General linear model with repeated measures was applied to assess the differences between the groups regarding the trajectory of glucose levels. Wilcoxon two-related samples test was employed to compare insulin doses at the start and at the 48th hour of treatment among groups. A p-value of <0.05 was chosen to represent statistical significance. Data were presented as median (25p-75p) or n (%).

## RESULTS

The study consisted of 76 children and adolescents [median age=10.0 (6.0-12.0); age range, 1.5-16.0 years; M/F: 44/32] with new onset T1DM admitting with DKA. Thirty six patients (47.4%) were pubertal. The median BG level on admission was 466 mg/dL (383-574) while the median BG level at the start of insulin was 158 mg/dL (123-198). Baseline characteristics of the study group are presented in Table 1.

Group 1 and Group 2 were comparable regarding age, gender, pubertal status, HbA1c, and BG levels both on admission and at the start of subcutaneous insulin treatment. Table 2 presents the descriptive data of the groups.

**Figure 2:**
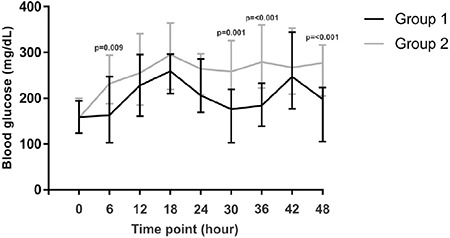
Median, 25^th^, and 75^th^ percentile values of the two groups at baseline and at specific time points during the first two days of subcutaneous insulin treatment. The p-values are given where statistically significant differences were present between the two groups

Both of the groups had similar numbers of BG measurements [Group 1, 8 (8-8) vs. Group 2, 8 (8-8)], p=0.250). Median BG levels of Group 1 in the first 48 hours were significantly lower than those of Group 2 [213 (171-242) vs. 255 (222-316), p=<0.001]. Figure 2 shows the trajectory of median BG levels during 48 hours and the difference between the curves was found to be statistically significant (p=<0.001). All median BG levels at specific time points in Group 1 were lower than those of Group 2, but statistical significance was present at 6^th^ [163 (103-247) vs. 232 (188-294), p=0.009], 30^th^ [176 (103-219) vs. 258 (178-326), p=0.001], 36^th^ [184 (139-233) vs. 279 (222-360), p=<0.001], and 48^th^ [198 (105-223) vs. 277 (205-316), p=<0.001] hours. Starting insulin dose (U/kg/day) and median glucose levels after starting subcutaneous insulin was found to be mildly correlated (r=-0.489, p=0.001) (Figure 1).

Rates of BG levels <50 mg/dL and <70 mg/dL were compared between the groups to evaluate the frequency of hypoglycemia, while the frequency of BG levels >200 mg/dL was evaluated in order to assess hyperglycemia (Table 3). Only two patients in Group 1 had experienced BG levels <50 mg/dL and those episodes were treated with oral glucose solutions. Frequency of BG levels <50 mg/dL, <70 mg/dL, and <100 mg/dL were similar in the two groups, while the percentage of BG levels >200 mg/dL were significantly lower in Group 1 (p=<0.001). The number of BG measurements in the target range (100-200 mg/dL) were significantly higher in Group 1 than in Group 2 (p=0.001).

The minimum and maximum BG levels during 48 hours were significantly lower in Group 1 than in Group 2 (p=0.004 and p=0.001) (Table 4). Table 4 presents additional glycemic variability indices of the two groups including BG rate of change, difference between minimum and maximum BG, SD and CV of BG.

During follow-up, subcutaneous insulin doses needed to be increased in order to avoid hyperglycemia. As a result, insulin doses (units/kg/d) administered on the second day in both Group 1 [1.63 (1.47-1.77) and Group 2 [1.07 (1-1.22)] were significantly higher compared to starting doses (p<0.001 for both).

## DISCUSSION

There are different approaches to initiate subcutaneous insulin after resolution of DKA, partly influenced by practice style and health care economics. In some institutions, basal-bolus insulin regimen is initiated immediately after resolution of DKA and the patient is discharged from hospital and further managed as an outpatient. However, many physicians prefer to start with subcutaneous regular insulin prior to discharge and determine the daily insulin requirement for the individual patient. Whichever is preferred, the primary concern is to maintain BG control while avoiding hypoglycemia. Early control of BG with insulin therapy might be associated with improved long-term glycemic control and higher endogenous insulin production (9,10). In practice, total insulin doses of 0.5-0.75 U/kg/day are typically chosen at T1DM onset and the dose is then adjusted on a daily basis to achieve the targeted glycemia. However, transition from 1.2-2.4 U/kg/day during treatment for DKA to much lower doses for subcutaneous insulin treatment often results in increase in BG levels, as was the case in the present study in both groups.

It is well-known that the metabolic abnormalities occurring in the diabetic state, in particular hyperglycemia, cause mitochondrial superoxide overproduction, which leads to activation of major pathways involved in the pathogenesis of complications of diabetes (11). Thus, the primary goal of treatment in children and adolescents with T1DM is to maintain near-normoglycemia as early as possible (12,13). In our study, the ratio of BG levels >200 mg/dL were significantly lower in Group 1 than in Group 2 (p=<0.001), a finding from which we may conclude that regular insulin at a dose of 1.4-1.5 U/kg/day prevents hyperglycemia better than the lower doses in the early period. In a previous study, it was reported that BG fluctuations were also associated with oxidative stress with coexistence of high BG levels and suggested that T1DM treatment should aim at reducing glucose fluctuations as well as achieving the overall control (14). In the present study, the difference between minimum and maximum BG, SD and CV of BG were similar in the two groups.

As a matter of fact, the issue that what is more important in the development of vascular damage is controversial in the literature. Peña et al (15) reported that hypoglycemia rather than BG fluctuation was correlated with vascular dysfunction in a pediatric population with T1DM. Moreover, several studies have shown that the most prominent barrier for tight glycemic control is the fear of hypoglycemia (16,17). In our study, there was no statistically significant difference between the groups with regard to hypoglycemia. In Group 1, only 2 patients had suffered <50 mg/dL hypoglycemia (each with 1 BG episode of <50 mg/dL) which was treated with oral glucose solutions. The number and ratio of BG <70 mg/dL and <100 mg/dL episodes were also similar in the two groups. None of our patients experienced severe hypoglycemia.

Wang et al (5) have recently compared the influence of different subcutaneous insulin infusion doses (0.6±0.2 U/kg/day, 1.0±0.2 U/kg/day, and 1.4±0.2 U/kg/day) on BG dynamics of children and adolescents with newly diagnosed T1DM and reported that approximately 90% of patients tolerated the higher insulin doses (1.4±0.2 U/kg/day) for 2 weeks without showing a significant difference regarding severe hypoglycemia rates. Our results are in line with those results and suggest that higher doses are necessary for better control in the early period. During follow-up, subcutaneous insulin doses needed to be increased in both groups in order to avoid hyperglycemia and the ratio of BG levels in the target range were only 37.5%, even in Group 1. Furthermore, the BG levels of the whole study group were only mildly correlated with initial insulin dose suggesting that higher doses could also be well-tolerated.

Late achievement of glycemic control can also result in increased hospital stay and thus, associated risks of hospitalization. However, due to the retrospective design of our study, we were not able to compare the duration of hospital stays in the two groups. Furthermore, there would be many other confounding factors that could affect hospital stay.

In conclusion, we suggest that an initial dose of 1.4-1.5 U/kg/day regular insulin may safely be used after resolution of DKA in children with new-onset T1DM with no increase in risk of hypoglycemia.

## Figures and Tables

**Table 1:**
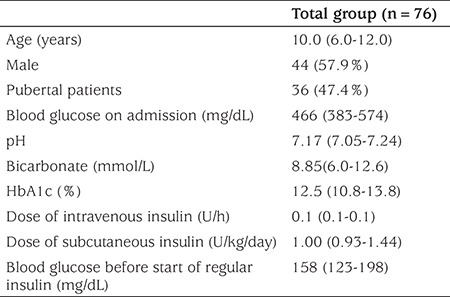
Baseline characteristics of the study group

**Table 2:**
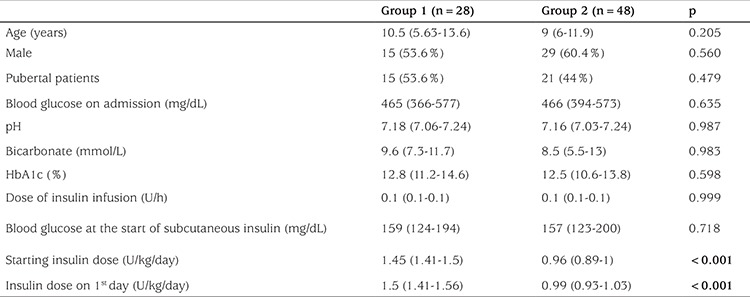
Descriptive data of patients among the two groups

**Table 3:**
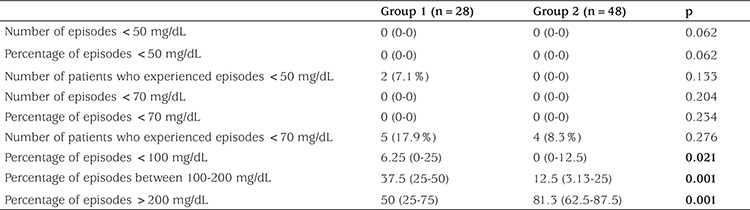
The characteristics of blood glucose fluctuations in patients with high-dose and conventional-dose subcutaneous regular insulin after resolution of diabetic ketoacidosis

**Table 4:**
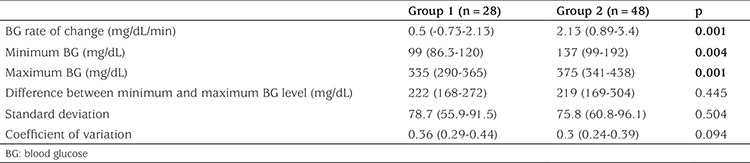
Glycemic variability indices among groups
